# Ethical dimensions of zoonotic disease research: Perspectives of traditional livestock keepers in Zambia

**DOI:** 10.12688/wellcomeopenres.17962.2

**Published:** 2023-06-12

**Authors:** Victor Chisha Zulu, Michelo Syakalima, Joseph Ali

**Affiliations:** 1 Clinical Studies, School of Veterinary Medicine; University of Zambia, P.O. Box 32379, Lusaka, Lusaka, 10101, Zambia; 2 Disease Control, School of Veterinary Medicine, University of Zambia, P.O Box 32379, Lusaka, Lusaka, 10101, Zambia; 3 International Health, Bloomberg School of Public Health and Berman Institute of Bioethics, Johns Hopkins University, Baltimore, Maryland, 21205, USA

**Keywords:** Livestock Keepers, Zambia, Zoonotic Disease Research, Research Ethics, Trust

## Abstract

**Background**: With the increase in zoonotic disease research using livestock belonging to traditional livestock keepers (LKs) as research subjects, careful attention to both animal and livestock keeper interests is critically important in Zambia and other similar contexts.

**Methods:** The study aimed to explore ethics-related challenges during zoonotic disease research among LKs where their livestock are included as research subjects. The study was implemented in the Southern province of Zambia in July 2020.  Three focus group discussions  (FGDs) involving 30 adult male LKs living in livestock-wildlife interface areas where zoonotic diseases are likely to occur, were carried out. The FGDs were done in the local language and audio recorded. Thematic analysis was done using field notes and translated and transcribed recorded interviews.

**Results:** The study found that trust between the researchers and LKs when their livestock are used as research subjects was very cardinal and depended on the continual presence of the local veterinary assistant (VA) during the conduct of research.

**Conclusions:** The LKs could be considered a vulnerable population when their livestock were used as research subjects as, being resource poor, they were looking to researchers to provide benefits yet not fully understanding the research, and thus did not worry so much about consent procedures, bringing into question the validity of the oral consent obtained. The study also found that opportunities to strengthen trust and enhance the research experience could be exploited by researchers conducting research that is locally relevant and desired, being aware of procedural preferences for entering into livestock keeping communities, adequate disclosure of research procedures, respecting conventions and traditional cultural beliefs, and returning results of research. The findings of this study can be used by both researchers as they carry-out zoonotic disease research and by Research Ethics Committees.

## Introduction

Zoonotic diseases are transmitted from animals to humans or are primarily endemic in animals but can infect humans as well. It has been observed that over 60% of diseases affecting human beings have their origin in animals ( Torrey & Yolken, 2005 in Babcock *et al.,* 2008). Emerging zoonotic diseases have often been the cause or suspected cause of epidemics and pandemics ( Jones *et al.,* 2008), such as the 2014–2015 Ebola outbreak in West Africa ( Calain & Poncin, 2015) and the 2019 Severe Acute Respiratory Syndrome Coronavirus 2 (SARS-CoV-2) pandemic ( Ciotti *et al.,* 2020; WHO, 2018). Because of the effects on the environment and settlement by human beings and their domestic livestock near wild animals, zoonotic diseases will continue to pose a health problem for humans internationally. Many zoonotic disease outbreaks occur in rural areas of low- and middle-income countries (LMICs), where traditional livestock keepers (LKs) live in close proximity to wild animals ( Malama *et al.,* 2014; Muma *et al.,* 2007). As such, an increasing amount of research on zoonotic diseases is being carried out in these areas, including in Zambia where many zoonotic diseases are endemic. Zambia conducts active surveillance of viral infectious diseases, and researchers are regularly investigating a a range of zoonotic diseases such as Brucellosis, Tuberculosis, Trypanosomosis, Anthrax, Leptospirosis, Rabies, Taeniasis, Toxoplasmosis, and Salmonellosis ( Dubey & Beattie, 1988; Hang'ombe *et al.,* 1999; Hobbs *et al.,* 2018; Laohasinnarong *et al.,* 2015; Malama *et al.,* 2014; Muma *et al.,* 2007; Munang’andu *et al.,* 2011; Ogawa *et al.,* 2015; Simulundu *et al.,* 2011; Sitali *et al.,* 2017).

As observed by Johansen and Penrith (2009), for cultural, social, and economic reasons, farmers attach great value to their livestock. For most pastoralists in Africa, their livestock represent significant value and can define their culture ( Ladbury *et al.,* 2017; Muma *et al.,* 2014). For some, possession of livestock is a source of pride, identity, and prestige in the community. Among the Maasai people (pastoralists of East Africa), cattle are viewed as being provided by God ( Ladbury *et al.,* 2017). In Zambia, traditional farmers, who are often monetarily poor, view their livestock as a resource to be used in times of need, such as when crops have failed. They are also viewed as a means of transporting farm produce, an engine for ploughing fields for crops, and as a medium of exchange for other essential family needs. Livestock can be used as gifts or payment in marriages and other social duties, like exchange for settlement of disputes, as well as for cultural ceremonies ( Muma *et al.,* 2014). High mortality rates of livestock due to diseases or other factors can evoke strong emotions of distress among those who own and care for the animals ( Mort *et al.,* 2008; Muma *et al.,* 2014). For these and other reasons, careful attention to both animal and livestock keeper interests when livestock are used in research is critically important in Zambia and other similar contexts. That zoonotic diseases may transmit from livestock to LKs also raises unique questions about the responsibilities of researchers and others. 

While there is now a significant amount of scholarship on the ethical dimension of research conducted with human beings, including in resource constrained settings, there is a significant gap in understanding expectations and obligations of researchers who conduct research with livestock. This void is especially apparent when considering the socio-economic and cultural importance of animals to many in regions where zoonotic diseases are endemic and research involving human and animal carriers is on the rise. To begin to help address this shortcoming, here we report findings of a qualitative study that explored the perspectives of traditional LKs in Zambia on the ethical conduct of research involving their animals. The paper explored whether the interests of the livestock keepers, whose socio-economic status was invested or dependent on their livestock, are given due consideration when conducting research using their livestock.

## Methods

### Ethical considerations

Ethical approval for the study was obtained from the Johns Hopkins Bloomberg School of Public Health Institutional Review Board (reference number 00009280) and the University of Zambia Biomedical Research Ethics Committee (reference number 009-02-19). Permission was also obtained from the District Veterinary Office, Ministry of Fisheries and Livestock, and from the local Chief in the study area. Oral informed consent and permission to audio record the discussions was obtained from each participant. Oral consent was considered adequate mainly due to the fact that the research procedures were minimal risk and the research ethics committee classified the study as exempt and approved the oral consent. The consent was documented by signing by the researcher obtaining consent.

### Study context

Zambia is a land-locked country located in Southern Africa, covering an area of 752,618Km ^2^. It has a population of approximately 16.5 million. The country is politically divided into 10 provinces, with three provinces (Southern, Eastern, and Western provinces) being the main livestock keeping areas. Cattle and goats are the main livestock, with Zambia having a total cattle population of over 3,700,000 and goat population over 3,500,000 ( Ministry of Fisheries and Livestock Central Statistical Office, 2018). The Southern Province was selected for the study as the people of Southern Province are traditionally cattle keepers, with a total provincial cattle population of over 1,300,000 ( Ministry of Fisheries and Livestock Central Statistical Office, 2018). For veterinary service delivery, a province comprises of several districts and is headed by the provincial veterinary officer; a district is divided in veterinary camps which are the smallest unit of veterinary service provision and is headed by the District Veterinary Officer. The veterinary camp is manned by the veterinary assistant (VA). LKs generally pay for veterinary services but the government veterinary services procure and provides vaccinations for free for diseases of economic importance during outbreak situations. The LKs trust the government veterinary system to help address their livestock problems. The study was implemented in Monze District, Southern Province, Zambia, in an area where LKs and their livestock live near Lochinvar National Park in close proximity to wildlife (Wildlife-Livestock Interface areas), and where zoonotic disease research has been carried out. 

### Study design

A cross-sectional, qualitative study was implemented. The design was used to capture the perceptions of LKs on the ethical dimensions of zoonotic disease research involving their animals. 

### Study instruments

Data was gathered through focus group discussions (FGDs) using a semi-structured guide that was conceptualized and developed by the researchers, with open ended questions to be able to obtain a deeper understanding of ethical and contextual issues surrounding zoonotic disease research among resource poor traditional LKs. The FGD guide covered the following broad topic areas: use of animals in research in general, general ethical issues, recruitment and informed consent process, awareness of zoonotic diseases, benefits and risks to self and livestock associated with research participation. Participants were also given a chance to add any other relevant information to the topic under discussion and to ask questions. The FGD guide was piloted in Central Province, Zambia to help improve clarity of questions and identify any substantive or procedural challenges. This was done with LKs that had previously participated in zoonotic disease research by invasive biological sampling where their livestock were research subjects, and living in a livestock-wildlife interface area. One pilot FGD was done with nine LKs. This led to clarification in the FGD guide on what is meant by the phrase ‘risks of research’, and an effort to prioritize certain questions to ensure discussions were not too lengthy. To this end risks were defined as ‘problems’ or ‘bad things’ ( Zulu, 2022b).

### Participants

The study included all adult traditional LLs living in livestock-wildlife Interface Areas where zoonotic disease research by invasive biological sampling for disease diagnosis, surveillance, prevention or control had been previously conducted with livestock and who agreed to participate. The LKs keep livestock traditionally as part of their culture and way of life. Purposive sampling was used, LKs were identified and invited to participate in the research by the local VA who knew those that had previously participated in research using their animals as research subjects.

### Data collection

Data were collected in July 2020 over three days through three FGDs across three different veterinary camps. The LKs were invited to come to one central place in their respective veterinary camps, where veterinary extension messages are usually communicated to them as a group. Usually this was at a community dip-tank. One of the authors experienced in qualitative research methodology, a native speaker of Tonga, the local language spoken in the study area, and well versed with the local customs, conducted the FGDs in Tonga using the FGD guide ( Zulu, 2022a; Zulu, 2022b). The FGDs lasted less than two hours each, were audio recorded, and field notes were taken by one of the researchers and two research assistants who were also native speakers of Tonga. To capture what the LKs were saying, credibility of responses was ensured by verifying responses from them. Names of the LKs were noted but during discussion the participants did not use their names to encourage confidentiality and anonymization of the data.

### Data management and analysis

The FGDs digital recordings were transcribed and translated into English by a researcher fluent in Tonga and English and crossed checked by another transcriber for completeness and accuracy. The recordings were then destroyed. Data was transcribed with no names of participants and stored on a computer protected with passwords. Field observations through expressions of participants, notes and transcripts were used in the analysis ( Zulu, 2022b). After each FGD, field observations were noted and considered for the next FGD if needed. Transcripts and notes were read several times by one study team member to begin to identify potential themes and codes through a combination of inductive and deductive processes – referring to question categories identified in advance within the interview guide, and other emerging topics within the transcripts ( Braun & Clarke, 2006; Creswell & Creswell, 2018). Several potential codes were identified and recorded in a codebook. The codebook was reviewed by all study team members over multiple rounds and with reference to transcripts. Following an iterative process, some similar codes were collapsed, and new codes were generated. Initially there were three broad themes identified - ethical conduct of research, motivation for participation in research and threats to ethical conduct of research. Later these were broken down to the following broad themes: LKs conception of research, identifying and approaching research participants, respect for participants, researchers and animals, motivations for LKs to participate in research, benefits, risks of participating in research, and disclosure and understanding of information about the research. Finally with further analysis and coding the following three broad themes were considered: LKs relationship with livestock, LKs conception of research and researchers, and trustworthiness between LKs and researchers during research. At all stages of analysis the broad themes identified had several codes below them. All data was coded manually. Quotations from participants were identified and selected for inclusion in this manuscript for illustrative purposes. The data was trust worthy seeing that similar perceptions were made across all the three FGDs. Data from the pilot discussion were not included in the analysis. 

## Results

A total of 30 LKs participated in the three FGDs (FGD1, FGD2, and FGD3, with 13, 10, and seven participants, respectively). While recruitment was gender-neutral, all participants reported being male as raising cattle is traditionally done by men in the study region. The livestock kept by the LKs included cattle, goats, sheep, pigs, and poultry. All participants ranked cattle as their most valued livestock, followed by goats. 

Broadly the study found that most issues raised by the LKs when zoonotic disease research was conducted with their livestock were rooted in the nature of their relationships with their livestock, the local VAs, and the researchers. Several dimensions of trust and trustworthiness emerged as both opportunities and challenges for zoonotic disease research, as described below.

### Relationship to livestock

All participants reported that they valued their livestock and attached great emotional and cultural importance to them. They considered cattle to be members of their family, gave them names, and talked to them. When their animals were sick, they suffered great emotional distress and felt like one of their own children was sick. They expressed that their livelihood depended on the livestock, without which life would be extremely difficult.

“Cattle are sources of livelihood- food, building houses, marriage, draught power, saved us during the drought, more like a bank.” [FGD3]
“Just like when my child is sick, cattle is part of the family, like a member of the family, have to find medicine to treat it and prevent it from dying. Feel bad like when a family member is sick when my cattle are sick.” [FGD1]

### General conception of research and researchers

LKs defined research generally as looking for information in a particular area. They considered researchers to be people who were foreigners or not part of their local community, who look for things that can impact their livelihood through their work on livestock diseases, which can affect agricultural policies. Research was also considered as collecting blood and other samples for the purpose of identifying or diagnosing diseases affecting their livestock. Most expected researchers to treat their livestock well during research, as if the livestock belonged to them, and were generally satisfied that researchers treated their livestock with respect.

“We think it means people who look for disease and other things on animals.” [FGD3]
“We expect good results as well as seeing the death rate of our animals decrease. We also endure with what they do and that gives us experience on how to work on our animals on our own. In addition to that, since they are educated officials, we believe they do the right thing to our animals.” [FGD2]

### Trust in researchers and research intermediaries (VAs)

Participants’ relationship with researchers was heavily dependent on trust. Researchers were held in high esteem by the LKs in general, and especially when accompanied by a local VA. Many participants found it difficult to refuse participation in research because they trusted that researchers, as experts, came to help them solve their livestock problems and that they came in good faith. While expressing some worry rooted in knowledge gaps, LKs agreed to participate in research because they thought they would miss out on benefits to their animals and also expressed that they would feel remorseful if they refused.

“Sometimes we worry because of ignorance since we do not have much experience on what vaccines they may give our animals but usually we have no worries on what they do since we trust them as our helpers.” [FGD3]
“We trust them as long as they introduce themselves and they come with someone from the District Veterinary Office.” [FGD2]
“We may think that we have done something bad [if refusal to participate in research].” [FGD2]

The local VA who lives and works in the community as a government worker providing veterinary and livestock extension services was key in the relationship between LKs and researchers. Participants depended on and put their trust in the VA as a gatekeeper and protector. The VA was considered to be someone who had the best interests of both the LKs and the animals at heart. The LKs needed an accountable agent to turn to should something go wrong, and the VA provided this function. All participants categorically stated that without the presence of the VA, they would not agree to participate in research. They trusted the VA, in part, because they always treated their animals in the community when they suffered from diseases, unlike researchers who rarely returned. However, even though the LKs wanted the presence of the VA, they were sometimes considered to be a hindrance to a direct relationship with researchers. Some of the LKs did not feel very free to discuss openly with the researchers in the presence of the VA, and they expressed that at times they perceived the VA as influencing their ability to seek more information or make an autonomous decision about participation in research.

“If no VA we don’t allow them [researchers].” [FGD2]
“We need someone local to complain to when things go wrong or when we need more information. This should be done even for subsequent visits.” [FGD1]
“Vet helps get the permission. We accept because the vet is our representative and is a government official.” [FGD3]
“Farmer should have the right of refusing and should be told. It becomes like a threat. We are not told to participate of free will. Because the VA was present, we agreed.” [FGD3]
“VAs don’t allow us to ask questions nor researchers to explain to us.” [FGD2]

### Opportunities to strengthen trust and enhance the research experience

Below is a description of subthemes that were identified as having an effect on the trust and trustworthiness relationship during implementation of research between the LKs and their livestock, researchers and the local VA. These activities were considered by the LKs that if implemented by the researchers would promote their participation in research. 

### Conducting research that is locally relevant and desired

Some participants expressed concern that researchers came with pre-planned research topics that do not address problems that LKs perceive as directly and imminently affecting them and their animals. This had a potential of hindering their continued participation in research.

“They should first listen to what we think/want before they do anything and then give advice.” [FGD1]

### Being aware of procedural preferences for entering into livestock keeping communities

Most participants expressed that before researchers approach them, they should first get permission from the District Veterinary office. Then researchers, along with the District Veterinary Officer and the local VA assigned to their camp or village, should seek permission from traditional leaders, usually the local Chief. The researchers, with the veterinary officers, should then approach the dip-tank committee for permission and help in identifying farmers who may be eligible and interested in research participation. The dip-tank committee manages the community dip-tank, which is used for application of acaricides on livestock, on behalf of the cooperative of LKs. The participants then expected the researchers, with the VA and the dip-tank committee members, to meet them as a group where all details and procedures concerning the research would be explained. Participants made it clear that if the above procedures were not followed, it had the potential of hindering participation in research.

“If when they come they don’t come through the Vet office and dip-tank committee, we will not welcome them.” [FGD1]
“[W]e expect them to agree with our local veterinary officials as well as the chief and our headman.” [FGD2]
“They should come through the committee so that we are informed and ask them questions together.” [FGD3]
“They should come with the vet or someone we know, otherwise we will not allow them.” [FGD3]

### Adequate disclosure of research procedures

A preference was expressed for researchers and others to meet with LKs as a group to share all details and procedures concerning the research. It was widely agreed by participants that researchers should explain the purpose of the research and research procedures to them. However, most LKs felt there previously had been inadequate disclosure of information on what researchers were going to do with their animals. This was compounded by their difficulty in understanding the research and its procedures. Some participants were also at times suspicious of what the researchers were doing, wondering if they implemented the agreed upon research procedures. They stated that the trust and confidence they had in researchers would be broken if researchers did not keep to agreed study procedures, such as the promised duration of research procedures. Some also expressed that they were not informed that they were free to refuse participation, though most felt that consent was obtained for research procedures. When asked what kind of consent they preferred, most generally expressed a preference for oral to written consent. Some felt that written consent may lead to loss of their animals.

“They explained before getting blood. They explained after introductions.” [FGD3]
“We sometimes think they do not come to cure since we may not fully understand what they come to do on our animals….They never explained what they were doing, we only agreed because they were with someone from the District Veterinary Office. We also agreed because we had expectations.” [FGD2]
“Teaching us what they want to do to our animals may be appropriate so that we have an open mind as we grant them to work on our animals.” [FGD2]
“Verbal permission is okay as long as you have talked and trust each other.” [FGD1]
“We don’t want to sign. Oral consent is the only thing done. No need to sign, we just need full disclosure and return of results. Signing may push us somewhere, we may sign for something that would lead to loss of animals.” [FGD2]

### Respecting conventions and traditional cultural beliefs

Some participants stated that they often handled and restrained their own livestock during sample collection and other research procedures, and that this made them feel respected by researchers. Others expressed that they would feel respected if researchers observed their traditional and cultural beliefs as they interacted with their animals during research. For example, a few participants stated that women are not allowed to touch bulls, believing that they may lose their reproductive capacity, nor to enter kraals where animals stay. Men mostly raised cattle and women were allowed and responsible for raising goats. If goats that were kept by women were the subject of research, it was believed that permission should be obtained from the husband, as the head of the household.

“We do not allow women at their monthly period from reaching the kraal and even ordinary women cannot visit the kraal side.” [FGD1]
“Permission only from the man as the head even if she keeps goats.” [FGD1]

### Returning results

Respondents were disappointed that researchers rarely returned results to them after their investigations with their animals. They also expected researchers to treat diseases that they found affecting their animals during research. They expressed that non-return of results of and not treating diseases affecting their livestock had the potential to compromise their willingness to participate in research. Some also thought that they could have done something wrong that made the researchers seemingly elect not to return pertinent information.

“We don’t see results, we see no benefit.” [FGD2]
“They just tell us what they are looking for. But they don’t tell us the results.” [FGD3]
“We may be left worried and wonder if there maybe any offence we might have committed for the official to leave without giving us any information because we expect some information about our animals and their health.” [FGD2]
“We expect a benefit of them telling us the problem he might have found on our animals. We also expect a benefit of having our animals treated from the diseases that affect them and since the government sends those people, we expect the government to send us the required vaccines for our animals if any problem is to mushroom at that time.” [FGD2]

## Discussion

This is the first study we are aware of in Zambia to explore perspectives of traditional LKs on the ethical dimensions of zoonotic disease research involving livestock. With increased occurrence of emerging and re-emerging zoonotic diseases, and consequently conduct of research in livestock-wildlife interface areas, the findings of this study are important to help researchers and those involved in research oversight appreciate the complexities of research conducted with livestock in many resource poor communities where livestock have deeply rooted cultural, economic and social significance. In such contexts, requirements related to the ethical conduct of research are likely to extend beyond traditional frameworks for governance of the research with animals, and perhaps even beyond frameworks for conduct of research with humans, given the relationship of dependency between human and animal.

The study found that when livestock under the care of traditional LKs in Zambia were used in research, trust between researchers and LKs was cardinal. LKs generally viewed researchers to be educated, knowledgeable animal experts who would be unlikely to cause harm to them and their livestock. Pearson and Paige (2012) noted that researchers also needed to build trust in communities when working with agriculturalists and pastoralists in Uganda in order to bolster participation in research. LKs in our study felt compelled to rely on researchers as caretakers of a highly valuable resource, and one that also was viewed as having intrinsic value. Participants expected researchers to respect their traditions, cultures, and community values, similar to how some communities in the neighboring country of Botswana have emphasized the importance of respecting such values when researchers conduct human subjects research ( Koloi-Keaikitse *et al.,* 2021). This is also in line with research ethics guidelines that emphasize the need to demonstrate respect for communities. 

LKs believed that researchers would recognize the value they placed on their livestock and treat both them and their livestock with respect while conducting research. However, the presence of the local village or camp VA who lived and worked among the LKs – at all times during research implementation – was cardinal to the relationship between LKs and researchers. The local VA was essentially viewed as a ‘trust broker’. LKs needed the active involvement of someone who was known to have previously sought to secure the best interests of their animals. Researchers were considered foreigners and could only be trusted with the presence of their local VA. Interestingly, the VA was also viewed as someone who could be held accountable for the actions of researchers, who were likely to not otherwise be accessible to the community. 

The study also found that LKs were more often than not willing to have their animals participate in research; however, certain stakeholder engagement procedures were critical. This included researchers seeking permission from the District Veterinary Officer at the district office and the local VA. This group, inclusive of the researcher, then needed to obtain permission from the local Chief, and then from the dip-tank committee. After this, the group was expected to meet the LKs, together, to explain what is being requested for purposes of the study. The dip-tank committee was effectively seen as a community advisory board, akin to what has been suggested in community-based human subject research, where similar boards provide a means for representative consultation with a community that is being approached for research ( Quinn, 2004). More generally, the above approach conforms with research ethics and guidelines that have documented and recommended community engagement as an essential procedure for ethical conduct of research ( Ahmed & Palermo, 2010; Council for International Organizations of Medical Sciences, 2016; National Bioethics Advisory Commission, 2001; Nuffield Council on Bioethics, 2002; Tindana *et al.,* 2007). The LKs’ preference for being approached as a group is also noteworthy, as this has the potential to increase comfort and control, perhaps offering greater opportunity to have their questions and concerns voiced and heard. 

Related, their limited power or ability to negotiate was demonstrated through finding it difficult to say no to research. In part due to their lack of resources, they were always willing to participate in research as they expected benefits and feared that not participating would mean that they would miss out on benefits accruing from the research and the government. In some contexts, LKs in Zambia may be considered to be vulnerable to exploitation and in need of additional protection as caretakers of animal research subjects ( Lange *et al.,* 2013). This is perhaps an atypical conceptualization of vulnerability in research; one that may be understood as a form of relational vulnerability.

Apart from recommending adherence to the above procedures and recognition of the positionality of LKs, the study found that researchers should consider how conditions that are the subject of zoonotic disease research are prioritized, how disclosure of study information takes place, and how results of research can be returned to participating communities for the benefit of LKs and their animals. Each of these are addressed in turn below.

According to Emanuel *et al.* (2000); Emanuel *et al.* (2004), and the Council for International Organizations of Medical Sciences (2016), research should seek to develop collaborative partnerships and be rooted in justifications that are both scientific and societal in nature. As such, research should seek to address a need within the community where it is being implemented, informed by processes of engagement with those who are most affected. LKs in Zambia are requesting more active participation in this regard. Generally, farmers keeping livestock are not considered major stakeholders when it comes to decisions regarding their livestock. This was also observed by Köhler-Rollefson *et al.* (2009) in India where for a long-time farmers had no input in the decisions made by the government regarding cross breeding of their traditional breed cattle with other breeds. In addition, despite being considered superior, it was likely that the other breeds lacked disease resistant capabilities that traditional breeds possessed. Köhler-Rollefson *et al.* (2009) further reported the birth of the ‘Livestock Keepers Rights’ movement for the conservation of local breeds. This movement has several declarations including “the right to be asked for prior informed consent (PIC), under the provisions of the UN Convention on Biological Diversity (CBD), whenever research is undertaken on their breeds or samples taken from them (these requirements tend to be systematically ignored by researchers and much research is of no practical relevance to livestock keepers)” ( Köhler-Rollefson *et al.,* 2009. p 1065). By coming with pre-planned research areas, it is in a sense perceived as overlooking the moral status of livestock keepers and their investment in the animals that they care for all hours of the day. While perhaps convenient to consider this type of research as ‘animal-subject research’, it is quite a bit more dynamic and draws attention to the livestock keeper-animal unit.

Further, our study found that despite participants having limited understanding of the technical and scientific research procedures, some still expressed that researchers did not adequately disclose information about the study, and that they were not given the opportunity to refuse participation. Interestingly, at times, even the trusted VA was seen as someone who could inappropriately influence the decision-making process. This made it difficult for LKs to know whether or not research was being implemented in conformity with approved and intended procedures. For informed consent to be valid, all essential information about a study should be provided in a manner that is understandable to decision-makers. Moreover, potential participants (and guardians) should be probed to confirm that they understand what is being communicated, and consent should be obtained freely and without undue influence ( Council for International Organizations of Medical Sciences, 2016). A preference for oral consent was stated by our participants, though this also means it will be less likely for there to be a record of consent having been obtained. While our data are limited here, written consent seemed be to less preferred possibly because it represented a method for exculpation – that is, written signed documents were perceived as having the potential to lead to giving up a right, for example, if something bad were to happen to the livestock as a result of the research. Written documents tend to convey a serious commitment that cannot be broken, and a means to protect the interests of those who are presenting the documents.

Ehsan *et al.* (2015), when studying the epidemiology of giardiasis – a zoonotic disease causing diarrhea in both cattle and humans – obtained written informed consent from farmers for collecting samples from them, and oral consent from farmers for collecting fecal samples from their cattle. Cooper *et al.* (2016) investigated the informed consent process obtained from traditional cattle keepers in Tanzania, simulating a research study for collection of blood and milk samples used in diagnosis of zoonotic diseases. Using three different strategies of presenting information to the LKs for consent, the researchers found that cartoons were the most effective followed by photographs. Written consent was identified as least effective. In contrast, Kaneene *et al.* (2017) obtained written informed consent to enroll both the farmer and their livestock as research subjects in Uganda. Variability in practice is not in and of itself problematic, but it suggests a need for further study to determine whether traditional criteria for determining the acceptability of alternatives to written, signed consent (such as research being of minimal risk) are the sole elements that need to be considered across all research contexts.

Finally, our study found a strong frustration among LKs due to non-return of research results by researchers. This has the potential to seriously compromise trust and dissolve the perceived value of research, hindering future participation in research by LKs. As observed by Knoppers and Lévesque (2011) and others in the context of human subject research, many researchers generally do not prioritize returning research results to participants and communities. Various structural and other factors interfere with and dis-incentivize the practice. In the context of zoonotic disease research, it was thought particularly important to LKs that they receive results from tests and other investigations with their animals to help them prevent and mitigate diseases that could ruin their livelihood. This potentially has implications not only for the health of animals, but also that of LKs themselves, who live in close proximity to their livestock and may acquire various diseases. Unfortunately, in some instances, LKs internalized these experiences and felt blameworthy, as if they did something wrong which resulted in their not receiving feedback from researchers. A similar sentiment was also expressed when our study participants reasoned about not wanting to refuse participation in research. Refusal was perceived as a wrong, and was guilt inducing. Unfortunately, we were not able to interrogate this further in this study, but it would benefit from further exploration in future research. 

### Limitations

A major limitation of this study is recall bias, as for some it may have been a while since their most recent engagement with zoonotic disease research. In addition, the study was limited in scope. It was largely exploratory, and as such was implemented only in the Southern Province of Zambia. The findings may therefore not be generalizable to other parts of Zambia, where zoonotic disease research is implemented among different ethnic groups. Further, no women were involved as participants in the study. Future research should seek to discuss this topic with women, who traditionally have had other roles in farming communities in Zambia.

Purposive selection of participants in this study could have led to selection bais in that the camp VA identified LKs who had previously participated in research. In future it would be better to also include LKs that had previously refused to participate in research.

In conclusion trust and respect are important when conducting zoonotic disease research among LKs using their livestock as research subjects. The importance of these two aspects were enhanced by the presence of the local VA when research was implemented. However, this may not hold under some political and social contexts where the LKs may not have confidence and trust in authorities. As reported in the study, the VA may also not be the best trust broker as reliance on the Dip tank committee and the Chief was also important in the procedures to follow before engaging the LKs in research. At minimum oral informed consent should be obtained and as a benefit to the LKs and their livestock, researchers should return research results and findings to the LKs.

### Recommendations

An ancillary, unexpected effect of their participation in our qualitative study was a feeling of empowerment among many LKs. They expressed that through their participation in our study, they felt more knowledgeable about research and expressed that going forward they knew more about what they had a right expect when research was conducted with their livestock. Educational interventions, for both traditional LKs and researchers, could be developed to build on this eagerness and to share expectations and concerns more widely. This could be done in coordination with relevant university, government, and community representatives.

Most of livestock in Zambia is still cared for in the traditional sector. Zoonotic disease research is usually done among LKs and their animals who live in close proximity to wildlife in livestock-wildlife interface areas. The findings of this study can be referred to by researchers before they carry-out zoonotic disease research, and by Research Ethics Committees (RECs) that review such research to ensure that research is conducted with full consideration of emerging ethical considerations. In addition, Institutional Animal Care and Use Committees (IACUCs) need to be established in Zambia, which can coordinate their reviews with RECs when research involves both animals and humans. The National Health Research Act (2013) in Zambia provides for the establishment and operation of RECs mainly to protect human participants in research, with mandate to also protect vertebrate animals in research, but there is no specific provision related to the establishment of dedicated IACUCs. This could be reconsidered, given the sometimes-specialized knowledge required for review or research involving animals. There is also a need for guidelines in Zambia for the use of agricultural animals in research. Again, these could be developed through a participatory process that engages all relevant stakeholders.

## Data Availability

Harvard Dataverse: Ethical Dimensions with Livestock Keepers during Zoonotic Disease Research. https://doi.org/10.7910/DVN/BX9XDB. ( Zulu, 2022a). The project contains the following underlying data: FGD1 field data.docx. (Record of the first FGD). FGD2 field data.docx. (Record of the second FGD). FGD3 field data.docx. (Record of the third FGD). Harvard Dataverse: Ethical Dimensions with Livestock Keepers during Zoonotic Disease Research- Data collection tools. https://doi.org/10.7910/DVN/XIAFBA. ( Zulu, 2022b). This project contains the following extended data: Farmer Focus Group Discussion Guide - English.docx. (Blank version of the guide in English) Farmer Focus Group Discussion Guide - Tonga.docx. (Blank version of the guide in Tonga) Data are available under the terms of the Creative Commons Zero "No rights reserved" data waiver (CC0 1.0 Public domain dedication).
