# Supplementary material for: Ethical dimensions of zoonotic disease research: Perspectives of traditional livestock keepers in Zambia
Source: Wellcome Open Res. 2023 Jun 12;7:201. Originally published 2022 Aug 3. [Version 2] doi: 10.12688/wellcomeopenres.17962.2 (PMC10366554; doi:10.12688/wellcomeopenres.17962.2)
Supplement: Peer review history

# Peer review: Reviewer response for version 2

**DOI:** 10.21956/wellcomeopenres.21593.r59937

**Published:** 2023-07-24

**Authors:** Samuel Ujewe

**Affiliations:** 1 Global Emerging Pathogens Treatment Consortium (GET-Africa), Lagos, Nigeria

The reservations raised in the earlier review have been sufficiently addressed by the authors.

Is the work clearly and accurately presented and does it cite the current literature?

Partly

If applicable, is the statistical analysis and its interpretation appropriate?

Not applicable

Are all the source data underlying the results available to ensure full reproducibility?

No source data required

Is the study design appropriate and is the work technically sound?

Yes

Are the conclusions drawn adequately supported by the results?

Partly

Are sufficient details of methods and analysis provided to allow replication by others?

Yes

Reviewer Expertise:

Research ethics, global health ethics, ethics of infectious diseases, African bioethics, cross-cultural bioethics

I confirm that I have read this submission and believe that I have an appropriate level of expertise to confirm that it is of an acceptable scientific standard.

# Peer review: Reviewer response for version 1

**DOI:** 10.21956/wellcomeopenres.19905.r51830

**Published:** 2023-02-13

**Authors:** Hayley MacGregor

**Affiliations:** 1 Institute of Development Studies (IDS), University of Sussex, Brighton, UK

**Overall comment:**

 This is a fascinating and timely study that provides very interesting and relevant findings and recommendations, despite the exploratory nature of the research. The study makes a convincing case that an approach to animal subject research that is concerned only with the welfare of livestock subjects does not appreciate the nature of the livestock keeper (LK) – animal unit/relationship and the dimensions of value (economic, cultural, emotional) that LKs assign to their livestock. In this regard, it is most timely to determine LKs’ understanding of the purpose and process of research as well as their expectations of researchers.

 The authors’ analysis points to the ‘relational vulnerability’ of the LKs, a term that captures well the tensions that can arise due to their economic precarity and the power relations at play that have potential to undermine informed consent. The central argument relates to trust and judgements of trustworthiness. In this regard, the findings reveal how the locally-employed Vet Assistants come to act as trust brokers/intermediaries in vouching for the researchers and providing government legitimacy to research endeavours which frequently involve foreigners. However, the quotes and analysis also suggest that the landscape of trust in such research is complex, with some ambivalence expressed also by the LKs about the extent of their autonomy and the intentions of researchers. For instance, responses from the LKs indicate that they have some misgivings about the consequences of refusing to participate in research. They also express misgivings about signing written consent and voice fears that this could lead to loss of livestock, presumably through expropriation. One issue that does not appear in the findings that I am aware has arisen in other projects relates to LKs expressing concerns that external people coming onto farms might potentially carry diseases. I wonder if this was raised at all by LKs and what might be the ethical considerations in this regard, particularly since animal research often is concerned with biosecurity on farms.

 The question of prioritisation of topics for research emerges as important and LKs express a desire for research relevant to their immediate concerns.  This finding is particularly pertinent given that emerging infectious diseases might not necessarily be seen as the most important issue to LKs, given that known endemic diseases are likely undermining productivity and causing mortality in the present. The authors make an important recommendation that dip tank committees should be consulted by researchers and could provide a stronger and collective voice for LKs to raise issues of concern, at the beginning of the research and in an ongoing way. The authors make a thoughtful link to wider literature on the use of community advisory boards in human subjects research and suggest an extension of this to research involving LKs and their animals, seen as an integrally intertwined unit.

 The responses of LKs in the focus groups also reveal potential disjunctures in understandings and expectations of the benefits of research. It is evident that the LKs expected vaccinations and other treatment, and certainly expected feedback of results of research and blood sampling pertaining to their animals and husbandry practices. It appears that this kind of feedback had not been a regular occurrence in their experience. What is left unclear in this manuscript is whether these LKs were given promises regarding treatments and vaccinations in the past that were unmet, and whether such ‘inducements’ are in fact regularly offered in the kind of research projects that they have had experience of. Perhaps it is the case that researchers might assume that the global health benefits of increased knowledge of EIDs offer sufficient wider societal benefits and do not routinely consider it necessary to offer immediate benefits to farmers in terms of knowledge or treatments. I am left a bit unclear as to how the expectations that are so evident in the quotes from LKs have arisen and what the routine practice in research projects might be. How significant then is the disjuncture between experience and expectation of LKs? Such a disjuncture might potentially affect judgements of the trustworthiness of researchers if promises have been left unmet.

 It would be useful if the authors could provide more information about recompense that might routinely be offered to LKs by research projects. I also wonder whether payments are routinely offered to Veterinary Assistants for the time taken to assist researchers. Such payments might on the other hand be seen to create conflicts of interest in the activity of recruiting LKs and acting as brokers, if LKs assume routinely that interventions are safe because VAs accompany researchers. It would be good if the authors could reflect on how such dynamics and the power relations underpinning interactions might include trust and recruitment if there are immediate financial benefits to VAs and indeed to the LKs recruited. A related methodological question for this study would be how such dynamics related to this research might have influenced recruitment, given that the researchers in this study also seemingly relied on the VAs to recruit participants for the FGDs. It would also be useful to know if a more concerted effort could have been made to seek the perspective of women - there is a suggestion that they do have some role in animal husbandry, at least with respect to goats. As the authors note, it would be important for future research to seek the views of women – there are some interesting statements about their agency in decision-making and it would seem important to seek their own perspectives.  I am aware that in some countries livestock keeping has been promoted recently as a source of economic and gender empowerment in agricultural policy (Ghana and Uganda come to mind).

 The authors reflect thoughtfully on the limitations of this study. It certainly seems as if this research has raised questions and laid the ground for a bigger study. In particular, a question does arise about how assumptions of trust might vary in political contexts where there is less trust in government and its agents than seems to exist in the region where this study was done. The authors briefly mention the possible effect of ethnicity but other forms of marginalization or state-citizen conflict might also have an effect on the extent of trust and the extent to which expert ‘scientific’ knowledge is taken as a sign of trustworthiness. It would be useful if the authors could just add a few lines to acknowledge that wider political and social contexts would affect trust and the implementation of the recommendations offered here. Can researchers assume that VAs are inevitably good trust ‘brokers’ for research?  How could a CAB approach ensure a wide range of participation of LKs in the local society, to ensure inclusive decision-making and equitable distribution of the potential benefits of research?

 These points aside, the recommendations and areas for future enquiry are very interesting and are raising important questions that fill a significant gap. The fact that some LKs expressed guilt, both in the instance of refusing participation in research and with respect to a lack of results feedback, is a significant element that would certainly benefit from further enquiry. Research in different socio-political contexts would also indicate new dimensions of relational vulnerability and other considerations regarding assumptions of trust. It would also be interesting in future research to juxtapose the understandings of livestock keepers with those of researchers and to explore forms of dialogue that might increase mutual understanding and advance more equal research collaborations with LKs.

**Specific comments:**

- **Background:**
  - In line with the last comment above, it would be useful to have just a little more context about the district where this study was carried out.
  - Are these pastoralists or sedentary livestock keepers? What is the extent and quality of government veterinary services and what is provided free of charge to farmers? What is the actual veterinary provision and do people trust the government veterinary system and believe it has their interests at heart?
  - I suggest that the authors also provide an indication early on of how they have defined research where livestock were used as research subjects. It would be useful to explain the scope of research under consideration in this study, from the authors point of view. Would it include qualitative research such as their own or was the concern here invasive biological sampling?
- **Methods:**
  - I do think some shifting around of text and information could improve the clarity of the methods section. For instance, does the ‘ *One FGD with nine LKs*’ refer to the one pilot FGD? For the main research, could the information be provided earlier about how many FGDs were conducted, in what geographic areas and how were LKs were selected.
  - The issue of gender balance is also only raised quite far down and some of the information about the FGDs is only provided at the beginning of the results section.
  - The clarity of this sentence could also be improved: ‘ *This was done among livestock keepers meeting the inclusion criteria for the main study, previously participated in zoonotic disease research’.*
* Participant selection: ‘….invited to participate in the research by the local VA who knew those that had previously participated in research using their animals as research subjects*’: I understand the reasoning here. However, it would be worth just reflecting on the potential limitations of this strategy. This could mean that these were people who trusted the VA and the vet system already or who the VA thought might give a positive view of research. It would be interesting in future work to also speak to a wider group of LKs, including those who might have refused participation in early research projects.
  - Location of research: ‘ *The LKs were invited to come to one central place where veterinary extension messages are communicated to them as a group*’: It would be useful to clarify this sentence. Is the implication that the research started with extension messages being relayed? Or that a site was chosen where this usually occurs? I also wonder if the VAs were present during the discussions as this might have limited what people felt comfortable to say, eg reporting negative experiences. The VAs might have been benefitting from payments for assisting research in general as well as this study, and thus concerned not to have negative reflections on livestock research.
  - Perhaps explain that the local language is Tonga for clarity.
  - The actual province or area has not actually been specified. Is there a reason for this?
- **Findings:**
  - Under section ‘Adequate disclosure’: ‘ *that researchers should explained the purpose*’  - change to should explain.
  - Under Discussion section: ‘ *potential to increased comfort and control*’ – change to potential to increase.

Is the work clearly and accurately presented and does it cite the current literature?

Yes

If applicable, is the statistical analysis and its interpretation appropriate?

Not applicable

Are all the source data underlying the results available to ensure full reproducibility?

Yes

Is the study design appropriate and is the work technically sound?

Yes

Are the conclusions drawn adequately supported by the results?

Yes

Are sufficient details of methods and analysis provided to allow replication by others?

Partly

Reviewer Expertise:

clinical medical anthropology

I confirm that I have read this submission and believe that I have an appropriate level of expertise to confirm that it is of an acceptable scientific standard.

# Response

**Published:** 2023-05-16

**Authors:** Victor Zulu

**Affiliations:** University of Zmbiaa, Zambia

We are thankful for the positive comments by the reviewer adding more value to help the findings of the study come out with clarity, thereby improving the presentation of the paper.  Some of the positive suggestions are actually areas of future inquiry and we greatly appreciate such positive observations.  

 There is a second part of the research where cross referencing will be done. This one involved obligations of researchers as they conduct this kind of research and will shed light on what they promise the LKs in terms of expectations or inducements. Otherwise the LKs just came out strongly that they expected results from the study to be given to them and participated so as not to lose out on benefits of government programs that are provided by the VAs. The LKs have these expectations as already alluded to in the manuscript because of their venerability. No compensation was given to the LKs for participating in the research. 

 Consultations with the veterinary department discouraged monetary compensations of LKs saying that such would hamper future animal disease control programs as LKs would be expecting monetary and other modes of compensation which the researchers could probably afford but not the government veterinary services. To this effect only refreshments were given during the FGDs. VAs were also only paid missing lunch allowance stipulated in their government conditions of service when they work in the field over lunch time. Few lines were added in the conclusion to offer clarity on trust that make be shaky under different situations political and social contexts.  As reported in the study the VA may also not be the best trust broker as reliance on the Dip tank committee and the Chief was also important in the procedures to follow before engaging the LKs in research.

 Most of the other suggestions and recommendations for changes are highlighted in the text and explained below.

 Responses to Specific Comments that needed further clarification:

*"In line with the last comment above, it would be useful to have just a little more context about the district where this study was carried out.* As highlighted in the text under study context, the study was done in Monze District of the Southern Province of Zambia near Lochinivar National Park. *Are these pastoralists or sedentary livestock keepers? What is the extent and quality of government veterinary services and what is provided free of charge to farmers? What is the actual veterinary provision and do people trust the government veterinary system and believe it has their interests at heart?"*

 Under study context and participants in the methods section more clarity was added. The LKs are pastoralists and traditionally or culturally keep livestock as a way of life. LKs generally pay for veterinary services but the government veterinary services procure and provides vaccinations for free for diseases of economic importance during outbreak situations. The LKs trust the government veterinary system to help address their livestock problems.

*"I suggest that the authors also provide an indication early on of how they have defined research where livestock were used as research subjects. It would be useful to explain the scope of research under consideration in this study, from the authors point of view. Would it include qualitative research such as their own or was the concern here invasive biological sampling?"*

 Research that was considered in the study was defined under study instruments and participants in the methods section as research that involved invasive biological sampling.

*"The clarity of this sentence could also be improved: ‘This was done among livestock keepers meeting the inclusion criteria for the main study, previously participated in zoonotic disease research’.*

*Participant selection: ‘….invited to participate in the research by the local VA who knew those that had previously participated in research using their animals as research subjects’: I understand the reasoning here. However, it would be worth just reflecting on the potential limitations of this strategy. This could mean that these were people who trusted the VA and the vet system already or who the VA thought might give a positive view of research. It would be interesting in future work to also speak to a wider group of LKs, including those who might have refused participation in early research projects."*

 This was discussed under limitations of the study “Purposive selection of participants in this study could have led to selection bais in that the camp VA identified LKs who had previously participated in research.  In future it would be better to also include LKs that had previously refused to participate in research.”

*"Perhaps explain that the local language is Tonga for clarity."*

 Under data collection in the methods section it has been explained that the local language is Tonga.

# Peer review: Reviewer response for version 1

**DOI:** 10.21956/wellcomeopenres.19905.r51832

**Published:** 2022-08-30

**Authors:** Samuel Ujewe

**Affiliations:** 1 Global Emerging Pathogens Treatment Consortium (GET-Africa), Lagos, Nigeria

The study sets out to explore potential ethical issues in the conduct of research on zoonotic diseases with livestock keepers in Zambia. Using a cross-sectional qualitative methodology with focused group sessions, it adequately captures perceptions of the livestock keepers with ethical implications. The study identifies a number of ethical issues related to research with Livestock keepers in Zambia, with prominent ones being Trust, consent, respect and benefit sharing, among others. The study concludes with a recommendation to develop educational interventions to both livestock keepers and researchers in relation to their rights and responsibilities within the research field.

 The study explores a gap in research ethics in Zambia, and much of Sub- Saharan Africa. It sets a pace for future exploration of ethical issues in research with animals owned by local livestock keepers, as it exposes a number of fundamental issues. Hence the study makes an original contribution to the knowledge and development of research ethics in Africa and globally. 

 Additionally, the methodology was described in sufficient detail to allow reproducibility of the research and the validity of the data used. 

 While commending the work, I would like to note that the presentation of the manuscript would need to be improved in two areas to make it more robust. From the introductory section, it is difficult to tell what the specific aim of the study/manuscript is, as no specific presentation of the ethical case is made and no explanation of the manuscript's goals is provided. I would suggest a revision of the introductory paragraph to provide for this shortfall.

 Secondly, the study results presents a number of important ethical issues which have been clearly outlined as findings. However, in the discussion section, the authors do not sufficiently engage the findings by way of a critical analysis or substantive ethical evaluation of the issues emerging from the study. For instance, the study results section describes emerging issues like trust, consent, conducting locally relevant research, respecting traditional cultural beliefs and returning results, among others. However, the discussion section largely provides additional explanations of the result as they emerged from the study. The issues of trust, consent and benefit sharing were lightly reviewed in the discussion, but not sufficiently engaged with to lead to a conclusive ethical statement on research ethics with animals owned by local livestock keepers. I would recommend that the authors revise the discussion section to critically review the ethical issues emerging from the results in a manner that leads to a sustained ethical conclusion.

Is the work clearly and accurately presented and does it cite the current literature?

Partly

If applicable, is the statistical analysis and its interpretation appropriate?

Not applicable

Are all the source data underlying the results available to ensure full reproducibility?

No source data required

Is the study design appropriate and is the work technically sound?

Yes

Are the conclusions drawn adequately supported by the results?

Partly

Are sufficient details of methods and analysis provided to allow replication by others?

Yes

Reviewer Expertise:

Research ethics, global health ethics, ethics of infectious diseases, African bioethics, cross-cultural bioethics

I confirm that I have read this submission and believe that I have an appropriate level of expertise to confirm that it is of an acceptable scientific standard, however I have significant reservations, as outlined above.

# Response

**Published:** 2023-05-16

**Authors:** Victor Zulu

**Affiliations:** University of Zmbiaa, Zambia

We are thankful for the positive comments by the reviewer adding more value to help the findings of the study come out with clarity, thereby improving the presentation of the paper.  Some of the positive suggestions are actually areas of future inquiry and we greatly appreciate such positive observations.

 To the first comment by the reviewer, the ethical issue and goal of the study was elaborated and summarized in the last paragraph of the introduction to show that the paper investigated the perspectives of the LKs regarding whether their interests were taken into consideration when their valuable livestock were used as research subjects.

 As observed by the reviewer in the second comment, we have reviewed the discussion regarding the issues of trust, consent and benefit sharing. We feel being the first of its kind or little information available on research ethics with animals owned by local livestock keepers it would be very quick to state board ethical conclusions. More empirical studies are required to come up with sustained ethical conclusions. However, to help highlight the findings of the study regarding, trust, consent preference and benefits, a conclusion statement was added to the discussion.
